# A Microscope Setup and Methodology for Capturing Hyperspectral and RGB Histopathological Imaging Databases

**DOI:** 10.3390/s24175654

**Published:** 2024-08-30

**Authors:** Gonzalo Rosa-Olmeda, Manuel Villa, Sara Hiller-Vallina, Miguel Chavarrías, Fernando Pescador, Ricardo Gargini

**Affiliations:** 1 Research Center on Software Technologies and Multimedia Systems, Universidad Politécnica de Madrid, 28031 Madrid, Spain; manuel.villa.romero@upm.es (M.V.); miguel.chavarrias@upm.es (M.C.); fernando.pescador@upm.es (F.P.); 2 Pathology and Neurooncology Unit, Instituto de Investigación Biomédicas I+12, Hospital Universitario 12 de Octubre, 28041 Madrid, Spain; sara.hiller.imas12@h12o.es (S.H.-V.); ricgargini.imas12@h12o.es (R.G.); 3 Pathology and Neurooncology Unit, Hospital Universitario 12 de Octubre, 28041 Madrid, Spain

**Keywords:** hyperspectral imaging, digital pathology, digital histology, whole slide imaging, microscopy, digital microscopy, biomedical imaging, hyperspectral biomedical, RGB imaging

## Abstract

The digitization of pathology departments in hospitals around the world is now a reality. The current commercial solutions applied to digitize histopathological samples consist of a robotic microscope with an RGB-type camera attached to it. This technology is very limited in terms of information captured, as it only works with three spectral bands of the visible electromagnetic spectrum. Therefore, we present an automated system that combines RGB and hyperspectral technology. Throughout this work, the hardware of the system and its components are described along with the developed software and a working methodology to ensure the correct capture of histopathological samples. The software is integrated by the controller of the microscope, which features an autofocus functionality, whole slide scanning with a stitching algorithm, and hyperspectral scanning functionality. As a reference, the time to capture and process a complete sample with 20 regions of high biological interest using the proposed method is estimated at a maximum of 79 min, reducing the time required by a manual operator by at least three times. Both hardware and software can be easily adapted to other systems that might benefit from the advantages of hyperspectral technology.

## 1. Introduction

Due to the increasing workload of anatomic pathology departments in hospitals around the world, along with a decrease in specialized pathology staff, outdated techniques, and the increase in technological investments, it is more necessary than ever to develop technological tools to help these departments work faster and more efficiently [1]. Nowadays, most hospitals around the world are integrating digital pathology to increase efficiency and accuracy in diagnosis and reduce human errors (known as inter-pathologist concordance) [2,3,4]. However, this implementation is not intended to dispense with healthcare professionals. Furthermore, the generation of public databases of digital pathology allows for the rapid propagation of transregional and international clinical data, taking advantage of the enormous advantages of biomedical image analysis using artificial intelligence.

Digital pathology offers the opportunity to work directly with high-quality images where pathologists can analyze the tissues and cells in much greater detail. These images are scans of either the entire sample or a significant part of it, which is commonly known as whole-slide (WS) imaging (WSI) [5,6]. In addition, digital pathology is constantly developing new deep learning-based tools for the automatic detection and analysis of cells and tissues, enabling faster and more robust analyses that allow for the study of previously unknown probabilities [7,8,9].

To capture these images, robotic microscopes called whole-slide scanners are used. Typically, these systems comprise a conventional brightfield microscope equipped with a transmittance lighting source, which may utilize various technologies such as LED, halogen, Xenon, or ultraviolet. Additionally, they feature a high-precision motorized plate, a camera capable of capturing the red, green, and blue (RGB) visible spectrum, and bespoke software that allows for the synchronization of all components to generate the images. Bueno et al. [10] presented an automatic system for capturing WS images in both brightfield illumination and fluorescence. This model also has an autofocus feature, which is a crucial functionality for this type of systems. Histological samples never have a flat surface, as the mechanical process performed to generate them produces micrometric variations on their surfaces. This means that when the sample is scanned, there are areas where a certain lack of focus is perceived because of irregularities in the tissue in that area. This is why WS scanners must have the ability to analyze the focus of what they are seeing at that moment and adjust the height of the platform to correct it [11].

Nevertheless, RGB technology leaves a great deal of useful information uncaptured, as it is limited to the visible range of the electromagnetic spectrum. For this reason, hyperspectral (HS) technology is one of the most promising technologies in the field of biomedical imaging [12,13]. Hyperspectral imaging (HSI) technology can capture spatial and electromagnetic spectrum information at the same time. Depending on the camera model, it captures a range of spectrum from visible (around 400 nm) to very near infrared (VNIR) (1000 nm) or even short-wave infrared (SWIR) (around 2500 nm). Currently, it is a well established technology in fields such as remote sensing [14,15], food quality analysis [16,17], and agriculture [18]. In the case of digital pathology, the advantage of HS cameras coupled in the aforementioned microscope systems that work with transmittance illumination is that when light passes through the tissue and reaches the sensor, the camera captures the spectral signature of each pixel without losing the morphological context of the sample. Given that tissues are highly heterogeneous, it is crucial to compress them both as a whole and according to their distinct characteristics. Therefore, when capturing a histological sample with different kinds of cell nuclei, membranes, red blood cells, etc., it is possible to study the independent signature of each element within the spatial context of the image. This allows for the development of classifiers based on machine learning or deep learning techniques that can automatically classify these elements.

In this context, the analysis of hyperspectral images in the field of digital pathology is beginning to prove beneficial. Meng et al. [19] proposed an analysis of membranous nephropathy samples captured with hyperspectral imaging through discriminant analysis based on spatial–spectral density peaks, obtaining satisfactory results. Ishikawa et al. [20] proposed a whole methodology for the analysis of HS images of pathological samples by decomposing the spectral signatures, while Maktabi et al. [21] proposed an analysis of different cells captured by HS images, in this case with a deep learning method. In the same line, Halicek et al. [22] proposed a technique based on the analysis of histological HS images for detecting carcinoma margins during resection time. Regarding recent acquisition systems, [23] proposed a system based on a hyperspectral setup integrating a snapscan camera with limited spectral resolution.

Despite these advances, hyperspectral microscopy remains far from being integrated into hospital pathology departments. Currently, limitations include a lack of technological standardization, the expense and difficulty of obtaining cameras, unavailability of complete acquisition systems on the market, the need for standardized preprocessing methods to generate the HS cubes used by camera and technology models [24], and the need for a reference approach to aid deep learning-based analysis.

To advance the integration and standardization of HSI in pathology departments, the first step is to standardize the acquisition systems. Therefore, the novelty of the work presented in this paper consists of introducing a microscope setup that integrates the following into a single system:

- The ability to capture, calibrate, and stitch hyperspectral cubes with a wedge-linescan camera in the range of 470 and 900 nm.
- The capacity to operate a high-resolution RGB camera configured for microscope use.
- An autofocus algorithm based on passive approach (i.e., by analyzing only the image capture by the sensor) that outperforms the ability of expert pathologists to focus the images.
- The capacity to create RGB whole-slide images with an algorithm that corrects possible errors in the overlap by applying affine transformations.
- An algorithm that allows for automatic capture of HS and RGB snapshots of areas designated by an expert pathologist. This functionality reduces the time that a system operator needs to spend performing the process manually by at least three times, in addition to ensuring perfect framing and reducing human error.

In addition to these functionalities, the following contributions complement the proposed setup:

- The creation of a simple labeling tool for whole-slide images that allows an expert pathologist to determine areas of biological interest to be captured in detail later.
- A methodology for using the entire system so as to ensure the correct capture of large databases in a fast and efficient manner. In addition, key features are determined in order to adapt the methodology to such a system.

The rest of the paper is organized as follows: Section 2 is divided into Section 2.1, where the hardware of the proposed system is described, Section 2.2, where the software development and implementation is described, and Section 2.3, where the methodology used to generalize and use the proposed system is described; all these functionalities are then validated in Section 3, with Section 4 including a final summary and future lines of research.

## 2. Materials and Methods

### 2.1. Microscopic Hyperspectral System

**Figure 1:**
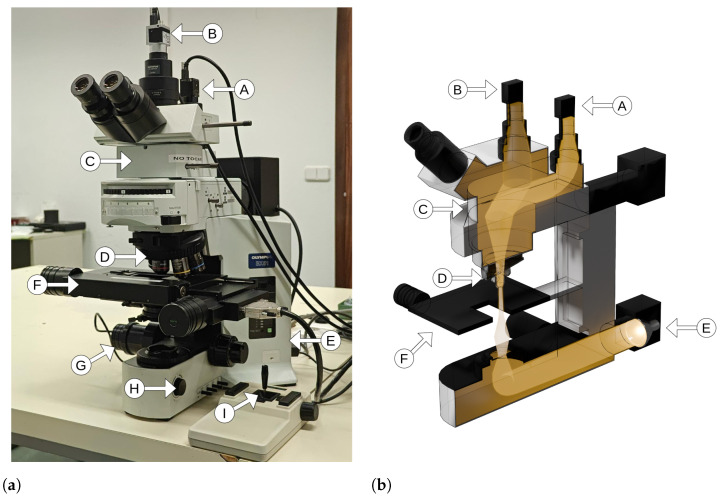
On the left of the image, a picture of the microscope hyperspectral and RGB system with its main components highlighted; on the right, an optical path diagram showing a simulation of the light traveling through the system. Components that can be observed include the following: (A) hyperspectral camera, (B) RGB camera, (C) trinocular tube, (D) lenses, (E) light bulb house, (F) motorized stage, (G) motorized Z-axis, (H) light power potentiometer, and (I) joystick. (**a**) Picture of the real microscope HS-RGB system and (**b**) optical path diagram.

The microscope RGB and HS capture system presented in this work is shown in Figure 1, and its specifications are detailed in Table 1. This device comprises an Olympus BX-51 (Olympus, Tokyo, Japan) brightfield microscope equipped with both a Basler ace acA5472-17uc RGB camera (Basler, Ahrensburg, Germany) and a Ximea LS-150 HS camera (Ximea, Münster, Germany). Both cameras share the same focus point thanks to an Olympus U-TRU trinocular intermediate tube attached between the main frame and the cameras. It is important to note that the light path selector knob of this trinocular is completely pulled out position; thus, only 20% of the light reaches the binocular and the RGB camera, while the remaining 80% goes to the hyperspectral camera. The values are imposed by the limits set by the manufacturer for this device. This decision is based on the fact that the hyperspectral camera has a lower quantum efficiency, meaning that more light power is needed to minimize noise in the final HS image.

The hyperspectral camera selected for this setup is a wedge-linescan camera with a CMOS (Complementary metal-oxide-semiconductor) 10-bit sensor. This wedge filter groups all the spectral bands in the range between 470 and 900 nm into a single frame. Its sensor has a resolution of 2048×1088 pixels, and each spectral band has a height of 5 pixels, making the theoretical number of bands 216. Nevertheless, according to the manufacturer 71 of these bands do not capture information, meaning that the number of useful bands is reduced to 145. As the bands are spread throughout the sensor, it is necessary to move the scene across the sensor to ensure that every band can capture all the points in that scene. This process is called hyperspectral scanning, and its result is hyperspectral raw data. The captured raw HSI data must be subjected to a series of operations, which is called the preprocessing stage, the result of which is a hyperspectral cube. The HS cubes captured by wedge-linescan cameras can have variable vertical resolution depending on the number of captured frames. The minimum resolution is always the same as the sensor; to retrieve it, it is necessary to capture twice the number of frames as the number of theoretical bands possessed by the sensor. In the presented system, this is 432 frames, as it has 216 theoretical bands, for a final resolution of 1080 pixels. However, if more frames are captured, then the vertical spatial resolution needs to be calculated according to Equation (1), where $R_{v}$ is the vertical resolution, *H* is the height of the sensor in pixels, $N_{f}$ is the number of captured frames, and $B_{h}$ is the height of a band in pixels. Therefore, the maximum vertical resolution is limited by the use case and memory restrictions.

(1) $$R_{v}=H+\left(N_{f}\times B_{h}\right)$$

**Figure 2:**
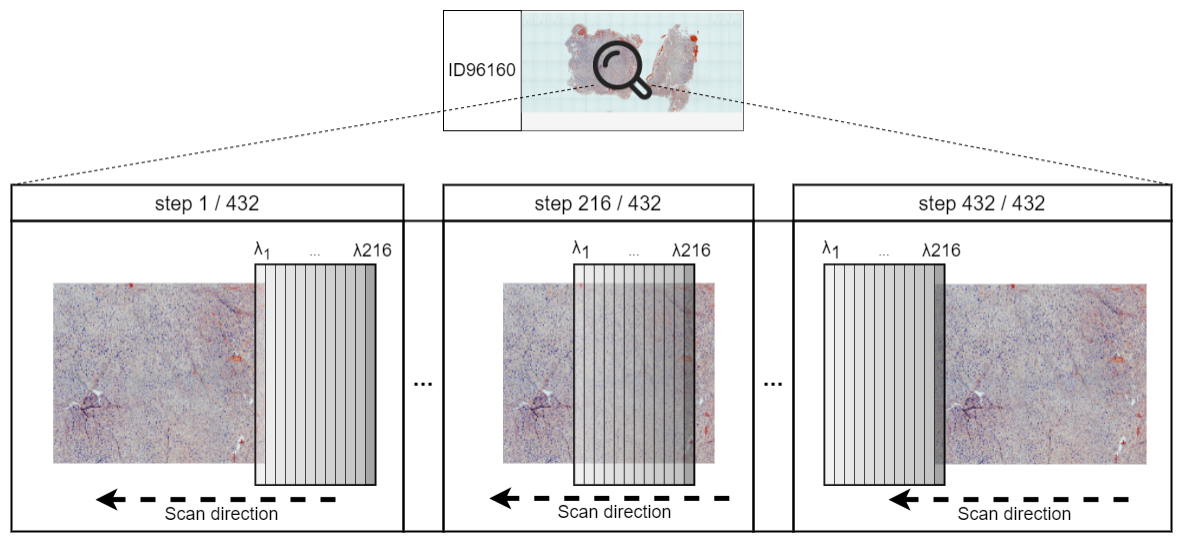
Diagram of a hyperspectral scan performed on an area of a histological specimen, showing how the camera slides along the entire scene in such a way that each band of the sensor captures each point of the sample.

Because the bands are arranged horizontally on the sensor, it is necessary for the scene to cross the sensor vertically in order to perform the HS scan. To do this, the HS camera is rotated 90 degrees with respect to the sample. Figure 2 illustrates this HS scan process from step 1, where the first band captures the beginning of the sample and starts to slide over the scene, through step 216, where half of the scene has been captured, to the last step, where the final band captures the last region of the scene. It is crucial that this HS scan is performed with adequate accuracy and speed to avoid deformations in the final capture. To ensure this, the motorized stage that integrates the sample holder has an accuracy of 0.02 μm/step. The whole HS scanning process is detailed in Section 2.2.1.

In addition to the motorized stage, the system has a Z-axis motor with a resolution of 0.1 μm/step that controls the height of the Z-axis stage. By controlling the height, the software of the system can automatically focus the cameras on the perfect focus plane, as described in Section 2.2.1. The X-axis and Y-axis motors, their encoders, and the Z-axis motor are all managed by a Prior H101BXDK driver, which is connected to the computer hosting the control application via a serial protocol.

The RGB camera of this system serves four purposes: to allow the system operator to easily navigate through the sample using a joystick connected to the motor stage driver, to focus the cameras, to capture a snapshot of a specific region, and to capture the frames that compose the WS scanning. This process is described in Section 2.2.1. The RGB camera is also placed in a 90-degree rotation with respect to the sample for visual and spatial matching with the HS camera.

This microscope HS and RGB capture system has three objective lenses: one with 4× magnification, one with 10×, and one with 40×. These lenses have transmittances of 95% in the visible range of the spectrum, with a decrease at 700 nm that ends at a transmittance of 50% at 1000 nm. Therefore, they cover the totality of the HS camera spectrum range.

Regarding illumination, the system includes a transmittance optical circuit without filtering, which is attached to a 100 W halogen bulb. The use of halogen technology is crucial to reduce the noise in the infrared range, as the spectrum of halogen builds is practically flat in the range between 500 and 2000 nm.

### 2.2. Software

As mentioned above, the microscope HS and RGB capture system presented in this work is able to automatically capture HS cubes in specific regions of a sample marked by an expert pathologist. In order to do this, three different software tools have been developed: the microscope controller, the labeling tool, and the HS preprocessing chain. Both the microscope control software and the preprocessing chain can be used by a non-specialized user; during this work, such a user is called the system operator. In contrast, the labeling tool must be used by an expert medical pathologist to ensure the veracity and judgment of labeled biological structures.

The whole software has been developed in a modular way in order to make it as easy as possible to adapt to other systems and components. As the final purpose of this system is to capture RGB and HS data to create databases, the main design criterion is that the system operator spends as little time as possible interacting with the system, ensuring at all times that the captured data are completely reliable. In addition, all of the code follows a modular criterion to reduce error and the operator’s interaction with the system as much as possible.

#### 2.2.1. Microscope Controller

**Figure 3:**
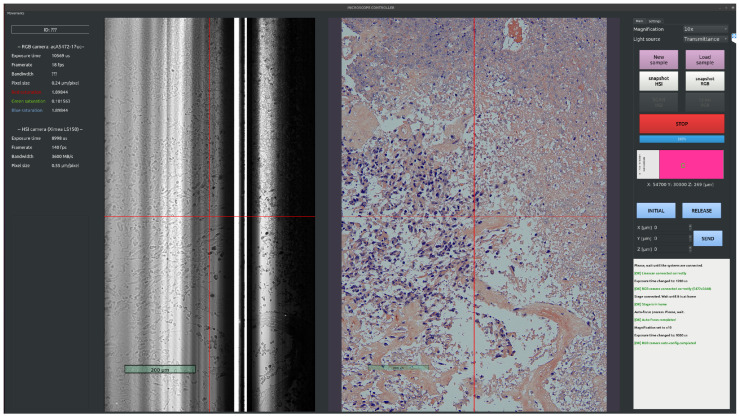
Custom GUI of the microscope controller.

This software is responsible for controlling all the devices that compose the system, synchronizing them to take captures, configuring them, and generating the image files. It has been developed in C++17 using the Qt 5.15 graphic user interface (GUI) framework. The GUI is shown in Figure 3. Its main features are:

- A. Autofocus of both cameras.
- B. Hyperspectral scanning (HS scanning).
- C. Whole slide RGB scanning (WS scanning).
- D. Automatic sequence of HS scans and RGB snapshots.
- Extra: Synchronization and managing the connected devices, both cameras, the motorized stage, and the Z-axis and to configure both cameras according to the selected magnification.

**A.**  **Autofocus**

**Figure 4:**
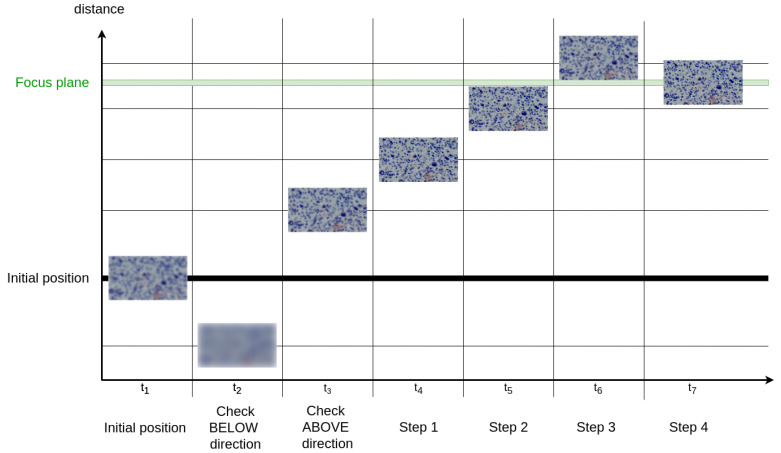
Diagram of the autofocus process. From the initial position, the system checks which direction the process is going in. To do this, it checks the position above and below the initial position. In this case, because the direction is uphill, it continues to move up until a the focus plane is found.

The autofocus feature is a functionality that adjusts the position of the Z-axis depending on the contrast evaluation of the RGB image in order to ensure the best focus of the image. To accomplish this, it performs a series of Z-axis movements followed by image evaluations, mimicking the natural movement patterns that an operator would use to focus the system. This approach is called ‘mountain-climbing’ [25]. In the system presented in this work, both cameras have the same focus plane, meaning that it is only necessary to evaluate the focus of the RGB images. This feature is performed automatically at both the start of the program and in the automatic sequence of HS scans; alternatively, it can be triggered manually by the system operator at any time. A diagram of the process is depicted in Figure 4, with the algorithm described below, where the default displacement distances depending on the magnifications are as follows: 10 μm for 4×, 5 μm for 10×, and 1 μm for 40×.

1. Disable manual controls (joysticks and keyboard shortcuts) to leave complete control to the software.
2. The stage is moved automatically to the default Z position, corresponding to the lens-to-sample distance specified by the lens manufacturer (also known as the working distance). In the presented system these are 470 μm for 4×, 350 μm for 10×, and 340 μm for 40×.
3. Take an RGB capture in the current position and evaluate its focus.
4. Next, it is necessary to evaluate the direction of movement (uphill or downhill). To do this, the Z-axis is first moved upwards by twice the default displacement distance, after which an evaluation of the focus of the frame is performed. Then, the same evaluation is performed by moving the Z-axis downwards by twice the default displacement distance from the initial position. If the evaluation is greater in the uphill direction, the Z-axis moves in that direction and the sequence of movements starts from that point. The same applies for the downhill direction. If there is no improvement in either direction, the Z-axis remains in the initial position, and the process ends.
5. If one of the two directions have been selected, repeat the following steps until the most focused image is found:
  (a) The stage is moved in steps of half the default displacement distance for the selected magnification.
  (b) In each step, an RGB capture is taken and the focus is evaluated.
  (c) If the evaluation of the focus is greater than the previous one, the loop continues. Otherwise, if the number of captures taken is less than 15, the loop continues. If the number of captures taken is greater than 15, evaluate all the captures and choose the distance where the focus is maximized. A maximum number of steps is specified to prevent the system of selecting false positives (local maxima).
6. When focusing is complete, re-enable manual controls and notify the system operator that the autofocusing is done.

The main goals of the autofocus feature is to perform it as fast as possible and while keeping the use of computational resources low. For this reason, and following the results presented in [11], we have applied a sharpening evaluation method that uses a Laplacian operator. The evaluation consists of converting the frame into grayscale, calculating its Laplacian using the OpenCV library [26], and calculating its variance. A high variance means that there is high spatial frequency, which also means that the borders in the image are well focused. It is important to note that the initial position from which this algorithm starts is equivalent to the optimal working distance of each magnification specified by the manufacturer. In this case, the initial distance is 470 μm for 4×, 350 μm for 10×, and 340 μm for 40×.

**B.**  **Hyperspectral Scan**

**Figure 5:**
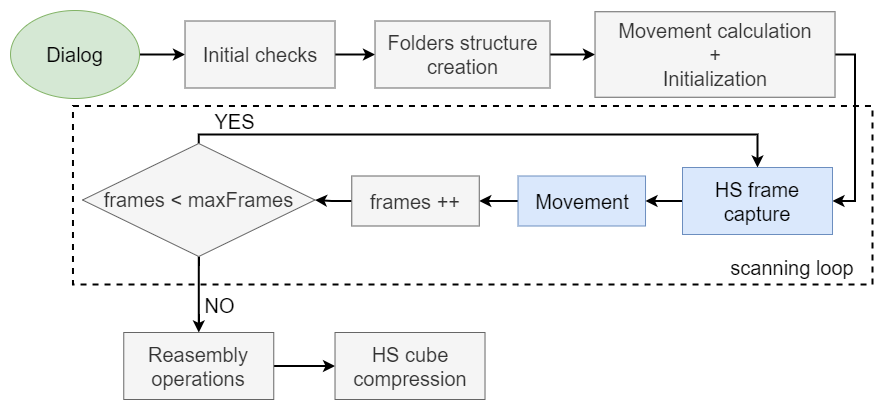
Hyperspectral scanning flow diagram. The blue boxes symbolize operations that involve interaction with hardware and the gray ones those that are solely software operations.

As mentioned above, HS scanning is the process of generating raw HS data by capturing frames from the HS camera while the sample moves along the sensor. A conceptual diagram of this process is illustrated in Figure 2. The raw HS data need to be preprocessed in later stages to convert the pixel information into useful spectral data. In order to perform the scan correctly, we have designed the algorithm represented in Figure 5. The steps are as follows:

- **User dialog**: A pop-up window where the system operator introduces the identifier (ID) of the sample, if the capture is for white reference or not, and the maximum number of frames to be captured during the scan. As explained in Section 2.1, the minimum number of frames is 432. From the number of frames introduced, the length to be covered by the scan is calculated based on the magnification set at that moment; thus, the system operator can determine whether the sample is fully covered.
- **Initial checks**: A check of every component of the system involved in the scanning process is carried out. If one of them is not operational, then the scan is aborted.
- **Folders structure creation**: This generates a folder in the work path with the sample’s ID; a folder inside it called *data*, which contains a folder with the magnification to be scanned.
- **Movement calculation**: In this step, the distance (*d*) that the motorized stage has to move between each frame is calculated by applying Equation (2), where $FOV_{v}$ is the vertical field of view (FOV) in μm of the HS camera (which depends on the selected magnification), $h_{band}$ is the height in pixels of a single band, and *h* is the height of the sensor in pixels.
  (2) $$d=\frac{FOV_{v}\times h_{band}}{h}$$
- **Initialization**: Disable the manual controls, create the scan information file, initialize the control variables, disable the open loop of the cameras to allow the algorithm to control when a frame is captured, and move the motorized stage half of the sample length to the left, which is the initial position for scanning.
- **Scanning**: This loop consists of capturing a frame, moving the stage the calculated distance *d*, and checking whether the step is the final one.
- **Reassembly operations**: Return the motorized stage to the initial position, enable manual controls, and enable the open loop of both cameras.
- **Compression**: This last step consists of grouping each of the captured frames into a single compressed file to save as much space as possible. Because each captured frame is ultimately a grayscale image and the scan is a succession of these frames, H.265 [27] lossless video compression with a ratio of 1.8:1 is applied to generate an video file in mp4 format. For instance, in a scan with 432 frames, the compressed file results in weights of 480 MB on average, while the uncompressed weight would be 920 MB. The compressed raw HS data are used in later stages for processing, as described in Section 2.2.3.

**C.**  **Whole-Slide Scan**

**Figure 6:**
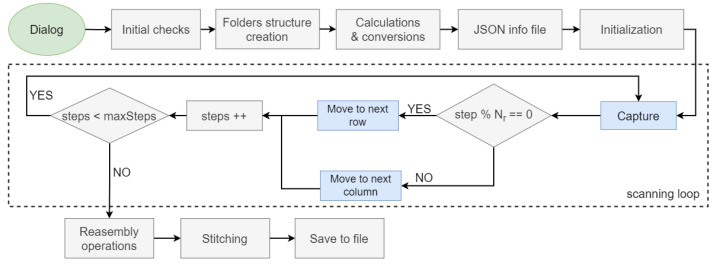
Whole-slide RGB scanning flow diagram. The blue boxes symbolize operations that involve interactions with hardware and the gray ones those that are software-only operations.

**Figure 7:**
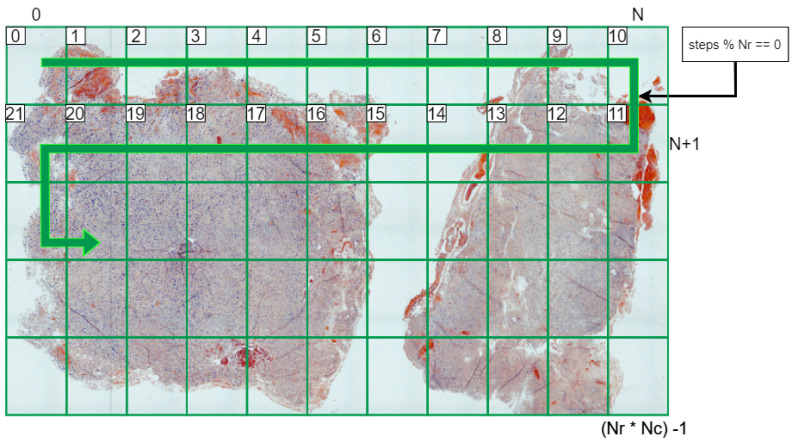
Whole-slide RGB scanning route.

As previously explained, the system presented in this work makes two contributions to traditional WS scanning. The first is to provide total freedom to the system operator in selecting an area to be scanned instead of the entire image, as the samples vary in size and shape from one another. This feature leads to significant savings in scanning time and disk space compared to the approach in which the whole sample is scanned regardless of its shape. The second contribution is a method for calibration and stitching of the captured images to create the final image through affine transformations based on a keypoint extraction by overlapping half of the image. The capture process is conceptualized in Figure 6, and its steps are as follows:

- **User dialog**: In this first step, the system operator determines the region to be captured using a pop-up window showing a simulated image of the sample. This step returns the coordinates of the top left point of the region ($R_{x}$, $R_{y}$) along with its width and height ($R_{w}$, $R_{h}$) in pixels.
- **Folder structure creation**: The folder structure used to save the created datafiles is created.
- **Calculations**: In this step, the number of images to be captured is calculated according to the size of the camera sensor. To do this, it is necessary to identify the initial point of the scan $\left(O_{x},O_{y}\right)$ in μm, calculated from the scanning region determined by the user. To transform from pixels to μm, Equation (3) is applied, where $TL_{i}$ are constants that represent the coordinates of the top left point of the sample (in μm), $BR_{i}$ are the coordinates of the bottom right point (in μm), and *W* and *H* are the width and height of the simulated image where the system operator has described the bounding box (in pixels).
  (3) $$\begin{array}{c} O_{x}=\left(TL_{x}-BR_{x}\right)\times \left(1-\frac{R_{x}}{W}\right)+BR_{x}\\ O_{y}=\left(TL_{y}-BR_{y}\right)\times \left(1-\frac{R_{y}}{H}\right)+BR_{y} \end{array}$$
  In addition, the coordinates $F_{x}$ and $F_{y}$ of the end point of the scan are calculated by applying Equation (4).
  (4) $$\begin{array}{c} F_{x}=\left(TL_{x}-BR_{x}\right)\times \left(1-\frac{\left(R_{x}+R_{w}\right)}{W}\right)+BR_{x}\\ F_{y}=\left(TL_{y}-BR_{y}\right)\times \left(1-\frac{\left(R_{y}+R_{h}\right)}{H}\right)+BR_{y} \end{array}$$
  With these two coordinates, it is possible to calculate the number of columns (number of images per row) $N_{c}$ and number of rows $N_{r}$ to be captured. To calculate these, Equations (5) and (6) are applied accordingly, where $FOV_{v}$ and $FOV_{h}$ are the FOV of the RGB camera corresponding to the magnification applied for that capture. It is important to note that the overlapping of the images needs to be half of the image to ensure that the stitching algorithm can merge the images correctly; this is why $FOV_{v}$ and $FOV_{h}$ are divided by two. Knowing $N_{c}$ and $N_{r}$, the total number of images $N_{T}$ can be calculated with the Formula (7). In this formula, note that the initial coordinates are referenced to the actuator and that the rows and columns take into account the rotation of the camera according to the actuator.
  (5) $$N_{c}=\frac{\left(O_{x}-F_{x}\right)}{\frac{FOV_{v}}{2}}$$
  (6) $$N_{r}=\frac{\left(O_{y}-F_{y}\right)}{\frac{FOV_{h}}{2}}$$
  (7) $$N_{T}=N_{c}\times N_{r}$$
- **Information file**: A JSON file is created with all the information about the scan.
- **Secondary operations**: Manual controls (joysticks and keyboard shortcuts) are disabled to leave complete control to the software. The stage is moved to the initial scanning position.
- **Scanning**: The loop consists of capture, saving into a temporary folder, and moving to the next position. If the last image taken is a modulus of $N_{c}$ of that row, move to the first next column of the next row (the one just below) and change the direction of the movement, i.e., by performing a snake scan pattern as shown in Figure 7.
- **Stitching**: This last step launches the stitching process described below to generate a final image with the final resolution of the WS image ($N_{c}$ × *H*, $N_{r}$ × *W*).

Because the parts that attach the cameras to the trinocular do not mechanically limit the camera to be rotated 90 degrees with respect to the sample, it is necessary to make this adjustment manually, which generates a certain error of some tenths of a degree. This error causes the scene to move along a diagonal line instead of a straight line when the stage moves in either dimension, resulting in misaligned overlaps between tiled images. Therefore, correcting this rotational error is essential for obtaining perfect image overlap. The stitching method proposed in this work corrects this non-constant error by applying an affine transformation matrix [28] with each image to be added to the final WS image. To carry out this process, it is necessary to calculate a matrix for when a capture is added horizontally and another for when one is added vertically. To calculate the matrices, the keypoints and descriptors of the overlapped zones are first obtained by applying the SIFT algorithm [29,30], chosen for its robustness and reliability. When the keypoints and descriptors between each image are obtained, they are matched by the brute-force algorithm [31], which has shown the best results for this application. The affine transformation is then calculated from the source and destination points obtained from the match. To exemplify this process, Figure 7 is used as a reference. The first step is to detect the keypoints and descriptors between each pair of images: 0-1, 1-2, 2-3, and so on, through 9-10. From these points, it is necessary to calculate the matrices between each pair of images that make up the entire row. Next, this process is repeated for each row. When each row has been generated, the keypoints and descriptors between each of them are detected, the matrices are calculated, and finally the whole image is composed. Depending on the image size, there may be limitations in terms of RAM or in the function’s own memory allocation; thus, it is recommended to divide each column into processing blocks for better efficiency.

In addition to applying its correction matrix to each image that makes up the final WS, it is necessary to correct the colors and illumination to achieve homogeneous lighting in the resulting image. To do this, each captured image is divided by a white calibration image, which is a capture of an empty slide at the same exposure time.

**D.**  **Automatic Sequence of Hyperspectral Scans**

**Figure 8:**
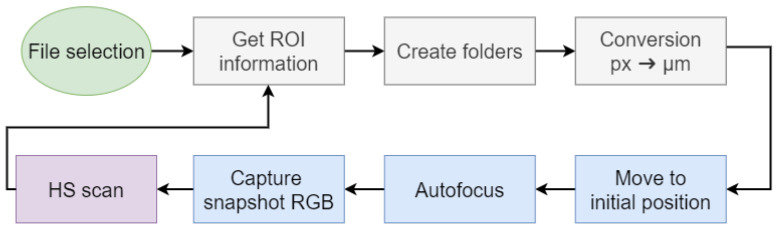
Automatic sequence of HS scans. The blue boxes symbolize operations that involve interaction with hardware and the gray ones those that are software operations only.

This functionality performs a sequence of HS scans in the ROIs labeled by the pathologist. First, the JSON file with the coordinates of the labels is read, then the JSON file with the WS scan information is read to retrieve the information of the scan coordinates. With all this information loaded in memory, the sequence loops through each labeled ROI and scans it as follows and it is summarized in Figure 8:

- **Get ROI information**: Read the information of the ROI (top left point coordinates in pixels, width and height in pixels, histopathology name, pathology number, and magnification used to capture the WS).
- **Create folders**: Create a folder with the name of the histopathology and its number.
- **Conversion**: In this step, the center point of the labeled ROI is calculated so that the center of the ROI matches with the center of the capture, then is converted from pixels to μm following Equation (8). In Equation (8), $O_{i}$ stands for the coordinates of the starting point of the WS scan, $ROI_{i}$ are the coordinates of the top left point of the ROI, $ROI_{w}$ and $ROI_{h}$ are the width and height of the ROI, *W* and *H* are the width and height of the sensor of the RGB camera, *M* is the magnification used to capture the WS image, and Px is the pixel size of the RGB camera (in μm).
  (8) $$\begin{array}{c} X=O_{x}-\left(\left(ROI_{x}+\frac{ROI_{w}}{2}-\frac{H}{2}\right)\times M\times Px\right)\\ Y=O_{y}-\left(\left(ROI_{y}+\frac{ROI_{h}}{2}-\frac{W}{2}\right)\times M\times Px\right) \end{array}l$$
- **Initial position**: Move the stage to the center position of the ROI.
- **Autofocus**: Perform the autofocus process to perfectly focus both cameras.
- **RGB capture**: Obtain an RGB snapshot capture of the centered region.
- **HS scan**: Start the whole HS scan of this region as described above. For these HS scans, it is sufficient for the number of frames to be the minimum number of frames, i.e., 432.

#### 2.2.2. Whole-Slide Labeling Tool

**Figure 9:**
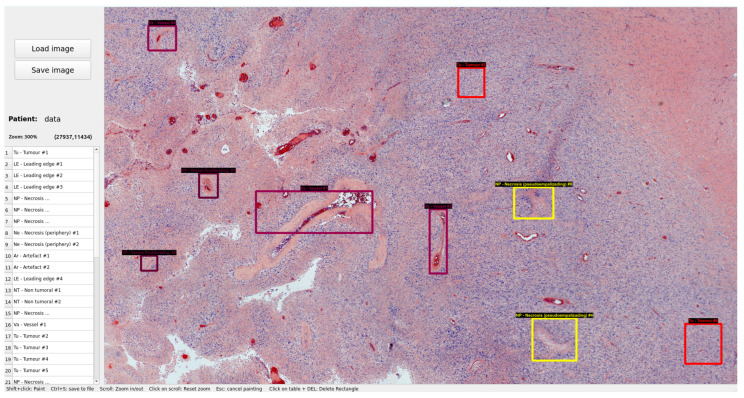
Custom GUI of the WS scan labeling tool. Each rectangle corresponds to a label and each color of each rectangle to a different pathology (tumor tissue, healthy tissue, vessel, edema, etc.).

When the whole-slide scan image has been captured using the software described in the previous section, an expert medical pathologist can analyze it to identify the areas of greatest biological interest. These areas can then be automatically scanned by the HS camera at higher magnification. This methodology is employed to avoid requiring HS scanning of the entire board, which would be completely inefficient in terms of disk space, time of capture, and processing time. The determination of biological structures is done by painting rectangular regions of interest (ROIs) without any kind of restriction in terms of the size or aspect ratio. The GUI of this tool is presented in Figure 9.

To use the tool, it is necessary to load a WS image. The pathologist can then navigate freely by dragging with the mouse to locate the most significant biological structures to be labeled and zoom in or out by scrolling. When located, the ROI must be drawn in such a way that it completely covers the structure while avoiding capturing adjacent areas as much as possible. Afterwards, the pathologist selects the histological area to which the ROI corresponds from a list. Each of the ROIs is stored in a dictionary that can be manipulated at any time by the tool to make corrections, delete any ROI, or sort them. The information stored in each ROI of that dictionary is as follows: the top-left point coordinates of the ROI, width and height of the ROI, histological area, and the number (e.g., the fourth tumor labeled in that WS scan).

The tool has been developed in Python using the GUI framework Qt 5.15, pyqtgraph 0.13 and OpenCV 4.9. Because the images have a very high resolution and are not compressed, it is necessary to run this tool on a computer with a minimum of 64 GB of RAM.

#### 2.2.3. Hyperspectral Preprocessing Chain

**Figure 10:**
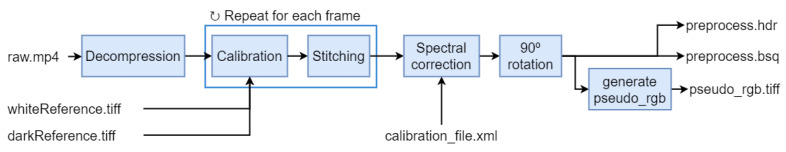
The hyperspectral preprocessing chain that converts compressed raw HS data into HS cubes suitable for processing.

After having captured all the HS raw data, it is necessary to process the data for conversion into useful HS cubes. The pipeline presented here performs this work in such a way that it leaves the HS data ready for future analysis. This process is called the preprocessing chain. The whole chain was developed in Python, and is described in Figure 10. Each stage of the chain is described below.

- **Decompression**: As explained above, after an HS scan the raw HS data frames are saved in a compressed lossless video format. In this stage, the video file is decompressed and each frame is loaded in memory.
- **Cube composition**: In this stage, each frame is calibrated and then fragmented band-by-band for placement in the final position. The stage is divided in two steps:
  – **Calibration**: In this step, radiometric calibration is applied to standardize the HS frame by referring the frame to two reference images that set the maximum amount of light (white reference) and noise level of the camera (dark reference) [32]. The white reference image is a capture of a non-sample area of the plate captured at the same exposure time with the same microscope adjustments as the frame to be calibrated. The dark reference image is a capture with the optical channel of the microscope covered; thus, the captured information is just the baseline noise of the camera. For calibration it is necessary to apply Equation (9), where R is the raw frame, DC is the dark reference frame, WR is the white calibration frame, and T is the transmittance calibrated frame. In a well calibrated frame, the transmittance values will be between 0 and 1.
    (9) $$T=\frac{R-DC}{WR-DC}$$
  – **Stitching**: This step places each band of each calibrated frame in its final position in the HS cube. The process is described in the work of Villa et al. [33].
- **Spectral correction**: When each band has been composed, it is necessary to multiply the whole cube by a matrix provided by the manufacturer according to the specifications of the camera. This orders the bands from least to most according to their wavelength and composes the spectral signature from the secondary lobes of other bands resulting from imperfections in the fabrication process of the camera filter [34].
- **Rotation**: As shown in Figure 2, the cameras of this system are oriented 90 degrees counterclockwise with respect to the sample to be captured. Therefore, in this stage it is necessary to rotate the entire cube 90 degrees counterclockwise to ensure that it correlates spatially with the sample. When this step is finished, the HS cube is saved in ENVI format [35] under the files “preprocess.hdr” and “preprocess.bsq”.
- **Pseudo-RGB creation**: In this last step, an RGB image is generated from the three bands closest to the pure bands of the visible spectrum colors (red, green, and blue). The purpose of this image, called “pseudo_rgb.tiff”, is to provide a visual reference of the captured image. In this case, the band selected for the blue color corresponds to 462.14 nm, the green one is 543.65 nm, and the red one is 605.61 nm.

### 2.3. Data Acquisition Methodology

In addition to describing the developed hardware and software, an associated methodology of operation is presented, along with all the configurable parameters that can be used to adapt this proposal to any brightfield microscope for generalization. Table 2 lists all the parameters that need to be evaluated in order to generalize the presented system in another microscope.

Taking into account the aforementioned hardware, software, and adaptability parameters, the following generalized methodology is proposed for capturing large quantities of histopathological samples in a fast and efficient manner:

1. **System checklist**: Before initializing the system, it is necessary to check the microscope from bottom to top, with no filters selected on the illumination optical circuit, the field iris diaphragm completely open, the condenser removed from the optical path. At top position, minor magnification above 2× must be selected, the knobs of the trinocular must be completely pulled out, the cameras connected, and the driver of the motorized stage powered on.
2. **Sample preparation**: After cleaning the protective glass of the sample with 90% isopropyl alcohol to avoid artifacts during capture, the sample is placed in the sample holder while ensuring that it is not twisted.
3. **Autofocus**: In this step, the microscope controller must be initialized; when the autofocus process is complete, it is important to check that the sample is well focused.
4. **WS scan**: The first image to be captured is the WS scan. To do this, it is necessary for the user to introduce the sample ID and then select the appropriate area to be scanned. In order to optimize the pathologist’s labeling time, it is recommended to capture the samples with the WS scan sequentially to allow the pathologist to label as many as possible in one working session.
5. **Labeling tool**: When the samples have been captured, the pathologist is notified to proceed with labeling. Thanks to the design of the labeling software, if the project is associated with a whole pathology department, the labeling process can be parallelized by dividing the samples into batches and assigning each batch to a different pathologist.
6. **Automatic sequence**: After labeling, the last step is to load each JSON file with the labels provided by the pathologist and then start the sequence of HS scans. In the capture sequence, the magnification required by the type of tissue to be captured must be selected. Normally, this is not the magnification used for the WS scan. The sequence of scans is fully automatic, and the system operator must change the sample when the capture is finished.

## 3. Results

In this section, a validation of the different critical points of the system presented in this work is performed, consisting of autofocus, the HS capture and preprocessing chain, and WS scanning. For this purpose, functionalities were tested using real brain tumor tissue samples from the cerebral cortex with wild-type Glioblastoma IDH (grade IV). These samples were taken at “Hospital 12 de Octubre”, Madrid, Spain.

### 3.1. Autofocus Validation

The first component to be validated is the autofocus system. For this purpose, a comparison between images focused by the presented autofocus algorithm and images focused by an expert pathologist was performed. To do this, the focus of the image was measured using the maximum variance of the Laplacian, described in Section 2.2, as a metric, then both variances were compared between each automatically and manually focused image. A total of 40 pairs of images (autofocused and manually focused) were captured, 20 for 4× magnification and another 20 for 10×. Of these pairs, ten were captured in random areas of tumor tissue with high heterogeneity and the other ten in random but very uniform areas of another tumor sample. The 4× magnification corresponds to the WSI and the 10× magnification corresponds to that used to capture brain cells.

**Figure 11:**
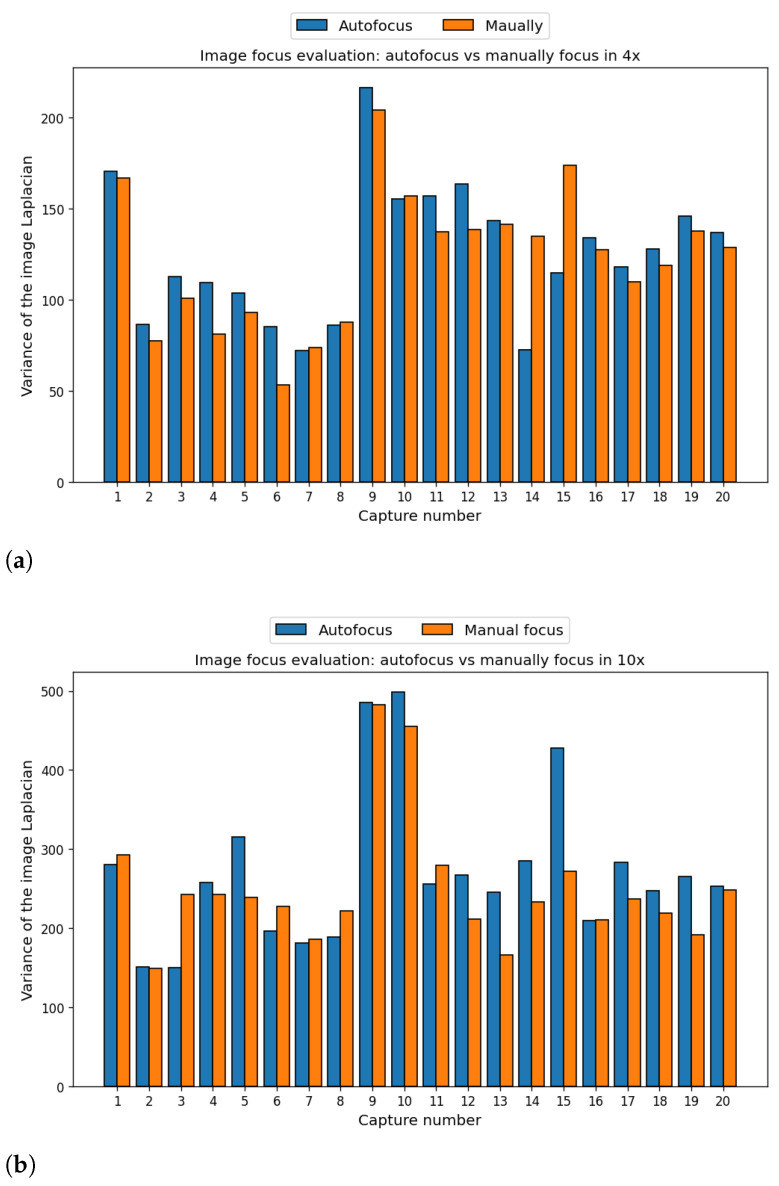
Focus evaluation comparison between autofocused and manually focused images. Figure (**a**) shows a comparison of images captured at 4× and (**b**) at 10×. The higher the value of the Laplacian, the higher the spatial frequency of the analyzed image, indicating better focus.

**Figure 12:**
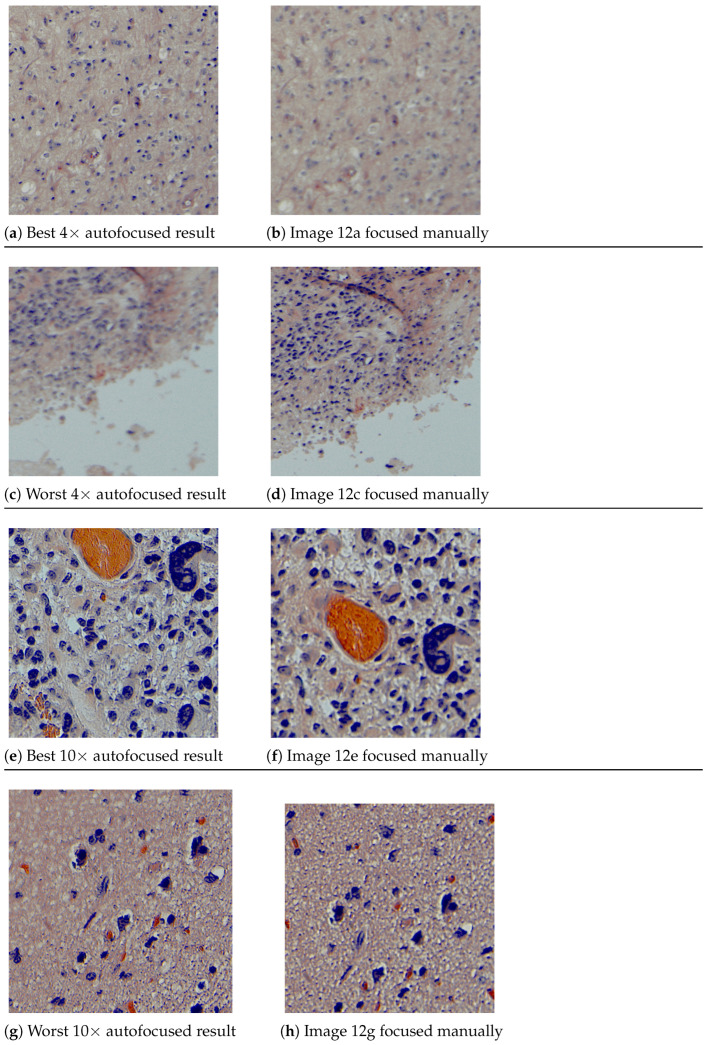
Central region (400 × 400 pixels) of the images focused by the autofocus algorithm and those focused manually: (**a**) is the best autofocused result in 4× compared to manual focus, with (**b**) corresponding to Figure 11a capture 12; (**c**) is the worst autofocused result in 4× compared to manual focus, with (**d**) corresponding to Figure 11a capture 14; (**e**) is the best autofocused result in 10× compared to manual focus, with (**f**) corresponding to Figure 11b capture 15; (**g**) is the worst in 10× is compared manual focus, with (**h**) corresponding to Figure 11b capture 6.

The comparison is shown in Figure 11, where the X-axis corresponds to each capture made in in different locations of each sample and the Y-axis to the variance of the Laplacian. In addition, Figure 12 shows the central region of 400 × 400 pixels for the best and worst results of the comparison between autofocused and manual-focused images. Quantitatively, i.e., by analyzing the metric used to evaluate the focus of the images, the autofocus algorithm outperforms manual focus in most of the images at both magnifications evaluated, although there are certain particular cases where manual focus is better. In capture number 14 of 4× (Figure 11a), there is a difference of 62 in the metric in favor of manual focusing. After analyzing the capture, it is observed that in this tissue location there is a fold produced during the sample processing that generates a height difference between the ridge of the fold and the tissue. The algorithm prioritized the fold, whereas the pathologist prioritized the tissue. Nevertheless, in the same capture 14 at 10× magnification (Figure 11b), a difference of 55 in the metric in favor of autofocus occurs, as the fold is no longer particularly visible and the autofocus algorithm is able to obtain improved focus. Another outlier occurs in capture 15 in 4× (Figure 11a), where manual focusing is better by a difference of 52 in the metric; there is a high concentration of red blood cells at this location, which generate a zone of higher energy causing the Laplacian to be maximized there. This causes this area of red blood cells to be more focused, leaving the rest of the image unfocused. Qualitatively, i.e., with the pathologist making a subjective assessment of the focus of the autofocused images, all captures are suitable for analysis with the exception of the aforementioned 4× capture 14. This capture can be evaluated in Figure 12e,f.

For the execution time, because the steps are sequential in a specific direction, the average time is 0.2 steps per second, including the processing time of each frame and the displacement of the platform.

### 3.2. Hyperspectral Scanning and Preprocessing Chain Validation

**Figure 13:**
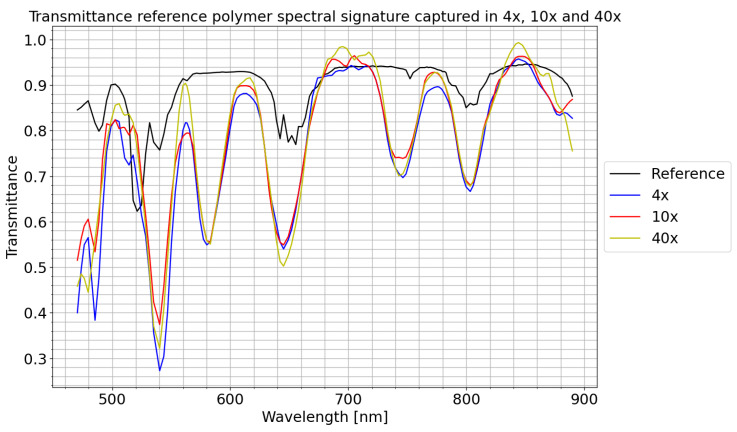
Comparison of the spectral signature of the reference polymer provided by the manufacturer against the spectral signature captured by the system presented at 4, 10, and 40× magnification.

To validate the correct operation of the HSI scanning functionality, a transmittance wavelength calibration standard was captured using the same scan configuration as the automatic scan sequence. This standard reference polymer, manufactured by Avian Technologies (New London, NH, USA), consists of a translucent surface with manufacturer-characterized wavelengths when halogen light passes through it. The same region of the polymer was captured at 4×, 10×, and 40× magnification with 432 frames, as can be observed in Figure 13, where the X-axis shows the wavelengths captured by the camera and the Y-axis shows the transmittance. After capturing the raw data, each raw spectral signature was prepared with the preprocessing chain, using a single frame of an empty glass (captured for each magnification) as a white reference image and a frame with the light turned off as a dark reference image.

As can be observed, the spectral signatures have very high correlation regardless of the magnification applied, showing that HSI scanning works for all magnification levels (Figure 13). Another important aspect that can be observed is the low correlation with the reference signature. The difference in amplitude between the three captures and the reference is due to the difference in the capture system and the calibration of both signatures. The difference in the form is because the reference spectral signature has been captured with a very high-precision spectrometer, resulting in the number of captured bands being six times the number of bands captured by the camera. In addition, the spectrometer has much higher quantum efficiency, making it better at detecting the peaks in the spectral signature of the material. However, the main characteristics remain the same.

In terms of execution time, the system takes 2 min 36 s to capture the 432 frames and 23 s (Intel processor i9-11900K @ 3.50 GHz) to compress them into a single file. The average disk weight of the file is 480 MB, while uncompressed it would be 920 MB.

### 3.3. Whole-Slide Scan

**Figure 14:**
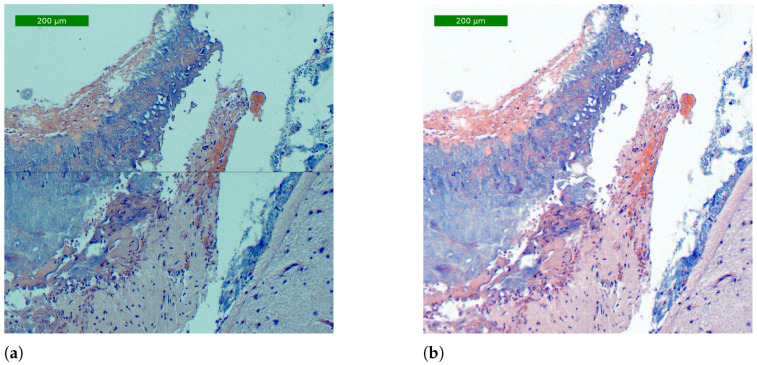
Crop of whole-slide images with scale resulting from application of the tiling and stitching methods: (**a**) detail of a WS image obtained with the tiling method and (**b**) detail of a WS image obtained with the proposed stitching method.

To validate the process, the result of an image generated by a simple tiling method and an image generated by the proposed stitching algorithm (described in Section 2.2.1) were compared. To capture the images for the tiling method, Equations (5) and (6) were modified to capture half of the image while ensuring no overlap between them. The final number of captured images was 28 (seven columns and four rows), with a capture time of 2 min and 30 s (5.3 s/frame). When captured, they were added one after the other in their final positions. To capture the images for the stitching process, the original values of Equations (5) and (6) were set, and the final number of images was 105 (fifteen columns and seven rows), with a capture time of 8 min and 30 s (4.82 s/frame). The whole stitching process required 2 min and 48 s. As can be observed in Figure 14, the result is clearly superior when using the stitching process, as the images are perfectly merged and the colors are closer to reality due to the calibration.

### 3.4. Use Case Analysis

**Figure 15:**
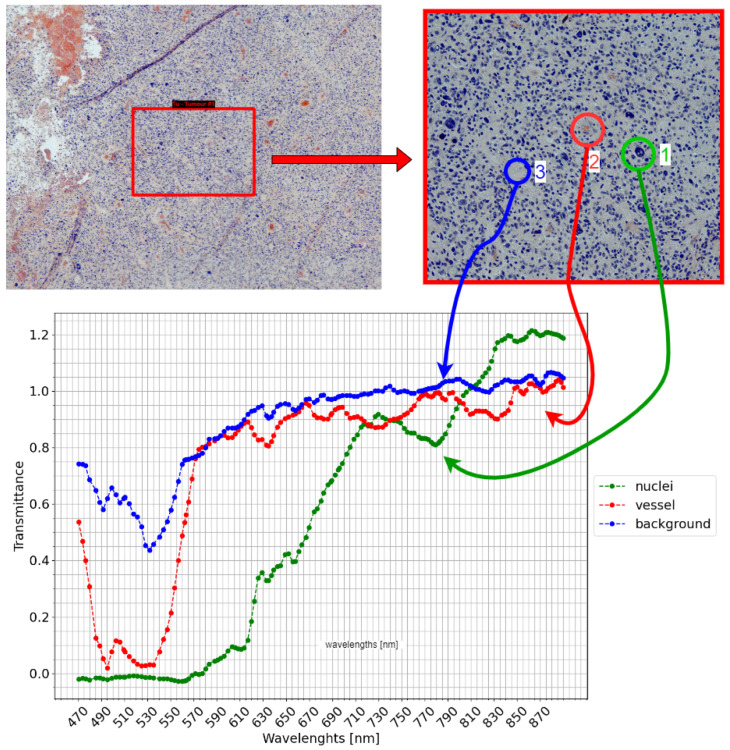
Reconstruction of each step of the entire experiment. In the top left image, the label of a tumor region assigned by the pathologist can be observed in the context of a whole-slide image. The top right image shows the region of the label captured by the HS camera at 10× magnification by the automatic sequence, and the bottom right image shows a plot of three significant spectral signatures of the centroid of a nucleus, a vessel, and the background.

Based on the obtained results, the entire methodology was then analyzed for application in a use case wherein a sample with twenty structures of high biological interest were captured. Following the methodology presented in Section 2.3, the system operator captured a WS image of the sample in 4× by scanning a region of 40,117 μm × 30,090 μm, which produced 105 images divided into fifteen columns and seven rows. When these had been captured and processed, the pathologist analyzed the WS image and then labeled all twenty zones within 5 min. After labeling, the system operator loaded the labels into the system and the automatic sequence of HSI scans was performed. Table 3 summarizes the total time for this entire process. As might be expected, the greatest time consumption occurs in the automatic sequence, taking up 60 min of the total 79 min. However, throughout this period of time the system operator does not need to interact with the system or supervise anything, as it is completely automatized. Of the 60 min corresponding to the sequence of captures, the automated displacement between label regions only requires a maximum of 20 s, while the remaining time is used for the actual hyperspectral scanning. In total, and depending on the morphology of the sample, it is estimated that performing this sequence manually would exceed the time taken by the automated sequence by at least three times. Table 4 summarizes the total disk space used for this experiment. All processing was carried out on a computer with an Intel processor (i9-11900K @ 3.50 GHz × 16) with 68 GB of RAM. Figure 15 shows the complete process in detail. In the figure, the image on the top left shows a region of the whole slide scan with a tumor label assigned by the pathologist. The top right image is the label captured by the automatic sequence at 10× magnification. From this capture, three spectral signatures have been selected: the centroid of the biggest nucleus, a vessel, and the background. As can be observed, the differences in the spectrum between each spectral signature are significant.

## 4. Conclusions and Future Work

The use of hyperspectral cameras in the field of digital pathology allows for in-depth analysis of the interaction of light with pathological specimens, while being able to study morphological properties thanks to their spatial and spectral capabilities. This work presents a microscope HS and RGB capture system for capturing histopathological samples easily and quickly at different magnifications with both RGB and HS technology, together accompanying control and processing software. In addition, the methodology designed to capture the samples correctly is fully adaptable to other HSI-RGB microscopy systems, and all of the steps are detailed in the paper.

This work combining a hardware system with control and processing software together with a clinical capture methodology represents a remarkable contribution to the field of hyperspectral imaging in pathology. In addition, the advantage of the presented approach is that it is easily adaptable to other types of microscopy, such as fluorescence.

From the validation we performed, the metric used to assess the autofocus system shows that the developed autofocus algorithm quantitatively outperforms manual focusing by an expert pathologist. During validation, it was detected that in tissues with a large number of red blood cells, the system prioritizes the focus on these red blood cells rather than on the tissue. The good performance of the focusing algorithm makes it very helpful when system operators need to capture samples manually, and also ensures correct captures when performing automatic sequences.

The generation of WS images has also been validated from the capture algorithm using a half-image overlap together with the stitching algorithm. Based on our experiences in developing this system, the use of the stitching algorithm instead of a simple tiling approach for creating whole-slide images is mandatory. This is because there is always a small drift in the alignment of the camera with the sample. In addition, the color and illuminance correction of each tile greatly improves the final image result, which facilitates subsequent labeling work by the pathologist.

Finally, the HS scanning performance has been validated, along with the automatic sequence of HS scanning and preprocessing. The automatic sequence of scans minimizes the time required for an experienced operator to perform capture by at least three times. In addition, it frees the operator from this tedious and error-prone task while ensuring perfect framing of the capture in the exact center of the label made by the pathologist. This functionality ensures that the captured regions are exactly as dictated by the pathologist. In addition, the preprocessing chain composes the cube bands correctly and corrects the spectrum to ensure that the spectral signatures can be analyzed easily and without the need for extra steps in future processing stages.

Based on the system described in this work, several measures that could be taken in the future to further optimize the system are listed below:

- Currently, the speed of the motorized stage is very conservative, which is to ensure the correct capture of the images in both the HSI scan and the WS scan to the greatest degree possible. Accelerating the speed of the capture system would reduce scanning times, which would be especially notable in the automatic sequence of scans.
- Accelerating the stitching algorithm to reduce the waiting time in generating WS images as much as possible.
- Applying more intelligent compression algorithms for the WS and HSI images could reduce disk size; this measure would be especially useful for capturing databases with a large number of samples.

## Figures and Tables

**Table 1:** Detailed specifications of the hyperspectral microscope system components.

System Component	Parameter	Value
Microscope	Model	Olympus BX51 (Olympus, Tokyo, Japan)
	Trinocular	U-TRU
	Lenses	UPlanSApo (4×) UPlanApo (10×) PlanApo (40×)
	Illumination type	Transmittance
Light bulb	Model	100 W 7724 (Phillips, Amsterdam, The Netherlands)
	Type	Halogen
	Spectral range	400 to 1800 nm
Motorized stage	Model	Stage X-Y PRior H101BXDK (Prior Scientific Instruments, Fulbourn, Cambridge, United Kingdom)
	Pitch screw X & Y axis	2 mm
	Resolution X & Y axis	0.02 μm/step
	Resolution Z axis	0.1 μm/step
HS camera	Model	MQ022HG-IM-LS150-NIR (Ximea, Münster, Germany)
	Camera type	Wedge linescan
	Spectral bands	145
	Spectral range	470 to 900 nm
	Sensor technology	CMOS
	Sensor resolution	2048×1088 pixels
	Band height	5 pixels
	Vertical FOV	4× = 6.875 μm 10× = 2.75 μm 40× = 0.687 μm
	Horizontal FOV	4× = 2860 μm 10× = 1144 μm 40× = 286 μm
	Sensor shutter	Global shutter
	Pixel bit depth	8 or 10 bits (configurable)
	Sensor size	11.3×6.0 mm
	Pixel size	5.5×5.5 μm
	Max. theoretical fps	170 fps
	Housing	C-mount
RGB camera	Model	Basler ace acA5472-17uc (Exton, PA, USA)
	Sensor technology	CMOS
	Sensor resolution	5472×3648 pixels
	Sensor shutter	Rolling shutter
	Pixel bit depth	10 or 12 bits (configurable)
	Sensor size	13.13×8.76 mm
	Pixel size	2.4×2.4 μm
	Max fps	17 fps
	Housing	C-mount

**Table 2:** List of parameters to be taken into account when adapting the proposed system to other microscopes.

Family	Parameter	Considerations
Cameras	Model	Olympus BX51 (Olympus, Tokyo, Japan)
	Camera rotation	If the camera(s) is 90 degrees rotated, the movement of the scans should be performed accordingly.
	Camera resolution	For quality, the minor resolution above 1920 × 1080.
	FOV spectral band	Transmittance
Calibration	White reference	Captures a frame of an empty and clean sample area.
	Dark reference	Capture a frame with the lighting system off.
	Spectral correction	If the HS camera requires it, apply according to manufacturer’s specifications.
Magnifications	Magnifications	Select the values according to the needs of the use case. Most common values: 5×, 10×, 20×, 40×.
	Whole slide scan magnification	The minor magnification above 2×.
	Z step	For the WS magnification, 10 μm. From there, scale it according to the ratio between the WS magnification and the desired magnification.
	Default Z position	Value equivalent to the working distance lens-sample specified by the manufacturer.
Ilumination	Technology	Recommended is halogen, but multi-LED spectral can also be used.
	Spectral range	It must cover the full range of the HS camera as well as the visible range for the RGB.

**Table 3:** Summary table showing the time required by each step of the entire experiment for 20 labels.

Action	Time [mm:ss]
Sample preparation time	1:00
Autofocus	0:30
Whole slide scan time	9:30
Whole slide stitching	3:20
Labeling time	5:00
Automatic HS sequence	60:00
Total	79:20 min

**Table 4:** Summary table showing the disk space occupied by the entire experiment: whole slide, labels, and automatic scanning of 20 labels.

File	Disk Space
WS image	1.2 GB
WS scan info file	338 bytes
Labels file	5 kB
Raw HS data compressed	480 MB × 20 scans = 9.4 GB
HS cube	2.7 GB × 20 scans = 54 GB
RGB snapshot	5.5 MB × 20 scans = 1.1 GB
Total	66 GB

## Data Availability

The original software comprising the methodology presented in the study is openly available in GitLab at https://gitlab.citsem.upm.es/public-projects/hyperspectral/hrgb-microscope-set-up (accessed on 23 July 2024).

## Abbreviations

The following abbreviations are used in this manuscript:

WSI	Whole-Slide Imaging
RGB	Red, Green, Blue
HS	Hyperspectral
HSI	Hyperspectral Imaging
IR	Infrared
ROI	Region of Interest
FOV	Field of View
ID	Identification
GUI	Graphic User Interface
CMOS	Complementary metal-oxide-semiconductor
